# The predictors to self-reported and performance-based physical function in knee osteoarthritis patients: a cross-sectional study

**DOI:** 10.3389/fcell.2024.1406830

**Published:** 2024-06-14

**Authors:** Qian-wen Wang, Gene Chi-wai Man, Ben Chi-yin Choi, Yi-man Yeung, Ji-hong Qiu, Xiao-min Lu, Michael Tim-yun Ong, Patrick Shu-hang Yung

**Affiliations:** ^1^ Department of Orthopaedics and Traumatology, Faculty of Medicine, The Chinese University of Hong Kong, Hong Kong, Hong Kong SAR, China; ^2^ School of Exercise and Health, Shanghai University of Sport, Shanghai, China

**Keywords:** muscle strength, performance-based physical function, predictor, knee osteoarthritis, self-reported physical function

## Abstract

**Background:**

Osteoarthritis (OA) knee patients have limited ability in physical function, or difficulties with physical tasks and activities may develop disability. This study aimed to observe the predictors of self-reported and performance-based physical function in patients with knee OA by analyzing the impacts of demographic, pathological, and muscle impairment factors.

**Methods:**

135 knee OA patients participated in this study to complete self-reported questionnaires using Knee Injury and Osteoarthritis Outcome Score (KOOS). When measuring performance-based physical function, a 6-meter gait speed (6MGS) test was measured to evaluate their mobility, and a 5-time Sit-to-Stand test (5STS) was assessed to evaluate their balance. Pain intensity, knee extensor and flexor muscle strength, age, body mass index (BMI), durations of symptoms, and radiographic severity were also collected. Spearman correlation and stepwise multiple linear regression were used to explore the association and predictors in self-reported and performance-based physical function.

**Results:**

BMI and durations of symptoms did not indicate any significant correlation with either self-reported or performance-based physical function. Age is significantly negatively associated with 6MGS (*r* ^2^ = −0.383, *p* < 0.01), while knee extensor muscle strength has a moderate correlation with 5STS (*r* ^2^ = −0.528, *p* < 0.01). In the stepwise multiple linear regression models, pain intensity (β = 0.712, *p* < 0.001), knee flexor muscle strength (β = 0.112, *p* = 0.042) were significantly associated with self-reported physical function in daily activities and contributed to 55.0% of the variance in KOOS-PF score. Knee muscle strength, including knee extensor (5STS: β = −0.428, *p* < 0.001) and flexor muscle strength (6MGS: β = 0.367, *p* < 0.001), were the main predictors with performance-based physical function.

**Conclusion:**

Pain intensity was the leading risk factor of self-reported physical function, and knee flexor muscle strength contributed as well. The severity of knee OA, durations of symptoms and BMI did not contribute to physical function. However, knee extensor and flexor muscle strength were the main predictors of performance-based performance. Our results show that strengthening of weak knee muscles in both quadriceps and hamstring muscle strength should be considered a priory consideration in knee OA no matter if people are in the early or end-stage of knee OA.

## Introduction

Osteoarthritis (OA) is one of the most common chronic aging conditions. The knee is the most frequently affected joint in the lower limb and contributes to significant mobility impairment. Up to 65% of people over 65 have radiographic evidence of knee OA (Baker et al., 2004). Knee OA is a significant cause of chronic disability in aging people (Guccione et al., 1994). Disability is the impaired performance of expected socially defined daily tasks in a typical sociocultural and physical environment (Jette et al., 2002). Therefore, limitations in physical function, or difficulties with physical tasks and activities, are fundamental to developing disability in OA.

Understanding the risk factors contributing to limited physical function is essential to improve functional limitations in people with knee OA. Two types of measurements are frequently used. One is self-reported questionnaires; some researchers may prefer them for convenience, saving time (Steultjens et al., 2001), and avoiding observer bias (Lingard et al., 2000). However, performance-based assessments, especially those that time activity or count repetitions within a specified time interval, may offer advantages over self-reported measures in evaluating change (Guralnik et al., 1989). In this study, we used self-reported and performance-based measurements because they measure different aspects of function and offer complementary information (Terwee et al., 2006).

Many studies investigated the risk factors for functional decline in knee OA patients, but most of them had patients with hip OA at the same time (van Dijk et al., 2009) and measured females only (Nur et al., 2018) or evaluated self-reported physical function only (Maly et al., 2006). However, clinical studies of physical functioning in knee OA have been lacking in evaluating the factors of physical function in patients with knee OA. Furthermore, there is a paucity of data in which demographic, pathological, and muscle impairment factors are integrated to detect the contribution of these potential risk factors to functional limitations. Therefore, this study aimed to observe the determinants of self-reported and performance-based physical function in patients with knee OA by analyzing the impacts of demographic, pathological, and muscle impairment factors.

## Materials and methods

### Study design

This cross-sectional study was conducted in compliance with the Declaration of Helsinki and was approved by The Joint Chinese University of Hong Kong—New Territories East Cluster Clinical Research Ethics Committee (Ethics approval number: 2021.491). This prospective study was conducted at the Prince of Wales Hospital, Hong Kong, from 1st June 2022 to 28th April 2023. Clinical diagnosis of knee OA was based on medical history and clinical examination of knee joints.

### Participants

In this study, 135 patients were admitted to our patient clinic and had knee OA according to the American College of Rheumatology criteria. The inclusion criteria for participants were listed as follows: 1) Knee OA patients over 50 years old clinically diagnosed with Knee OA with KL grade 2 or above; 2) Able to walk unaided for 6 m; 3) Both knees without a history of injury/prior surgery. In addition, participants were excluded if they had connective tissue disorders or myositis conditions and a history of cancer. Written informed consent has been obtained from all participants involved in this study.

#### Dependent variables (outcomes)

##### Self-reported physical function

Physical function was measured using the KOOS function in daily living (ADL) subscale for the physical function outcome. The KOOS is a commonly used patient-reported outcome with overall acceptable psychometric properties to evaluate patients with a knee injury and knee OA, including those having TKR (Roos and Toksvig-Larsen, 2003). KOOS contains five subscales with a total of 42 items: 1) pain; 2) other symptoms; 3) function in daily living (ADL); 4) function in sport and recreation (Sport/Rec); and 5) knee-related quality of life (QOL). Each question receives a score from 0 to 4, and the scores are transformed to a 0 to 100 score (0 = extreme symptoms, 100 = no symptoms). The reliability and construct validity of KOOS has been extensively demonstrated in recent years (Collins et al., 2016).

##### Performance-based physical function.

###### 6-meter gait speed tests (6MGS)

The six-meter gait speed test was used to measure the mean walking speed (in seconds) after three walking attempts for 6 m along a straight line. Participants were informed to walk 6 m at their usual speed. Gait speed measurement were calculated: meter (6 m)/time (seconds). 6MGS at a normal pace is a reliable assessment for estimating physical function in chronic disease aged adults including knee OA patients (Kim et al., 2016).

###### 5 time sit-to-stand tests (5STS)

The five-time sit-to-stand test (5STS) is a physical performance test commonly-used in clinical geriatric studies (Bohannon, 1995; Whitney et al., 2005). This test is easy to perform in clinical practice and has shown excellent intra- and inter-rater reliability (ICC, 0.89) in patients with severe hip or knee OA (Villadsen et al., 2012). Patients were asked to sit on a solid chair with arms on shoulders and feet shoulder-width apart. They were asked to perform five repetitions as quickly as possible.

#### Independent variables (predictors)

##### Demographic data

Demographic data were recorded with the first screening appointments. Age, durations of symptoms, height, weight, and body mass index (BMI) were recorded. Body weight and height were measured using a standard stadiometer, and the body mass index (BMI) was calculated (body mass in kg/[height in m]^2^). In brief, Participants removed their socks, stood on two metallic electrodes on the floor scale barefoot, and held two metallic grip electrodes placed in the palm of their hand with their fingers wrapped around the handrails.

##### Muscle strength

**FIGURE 1:**
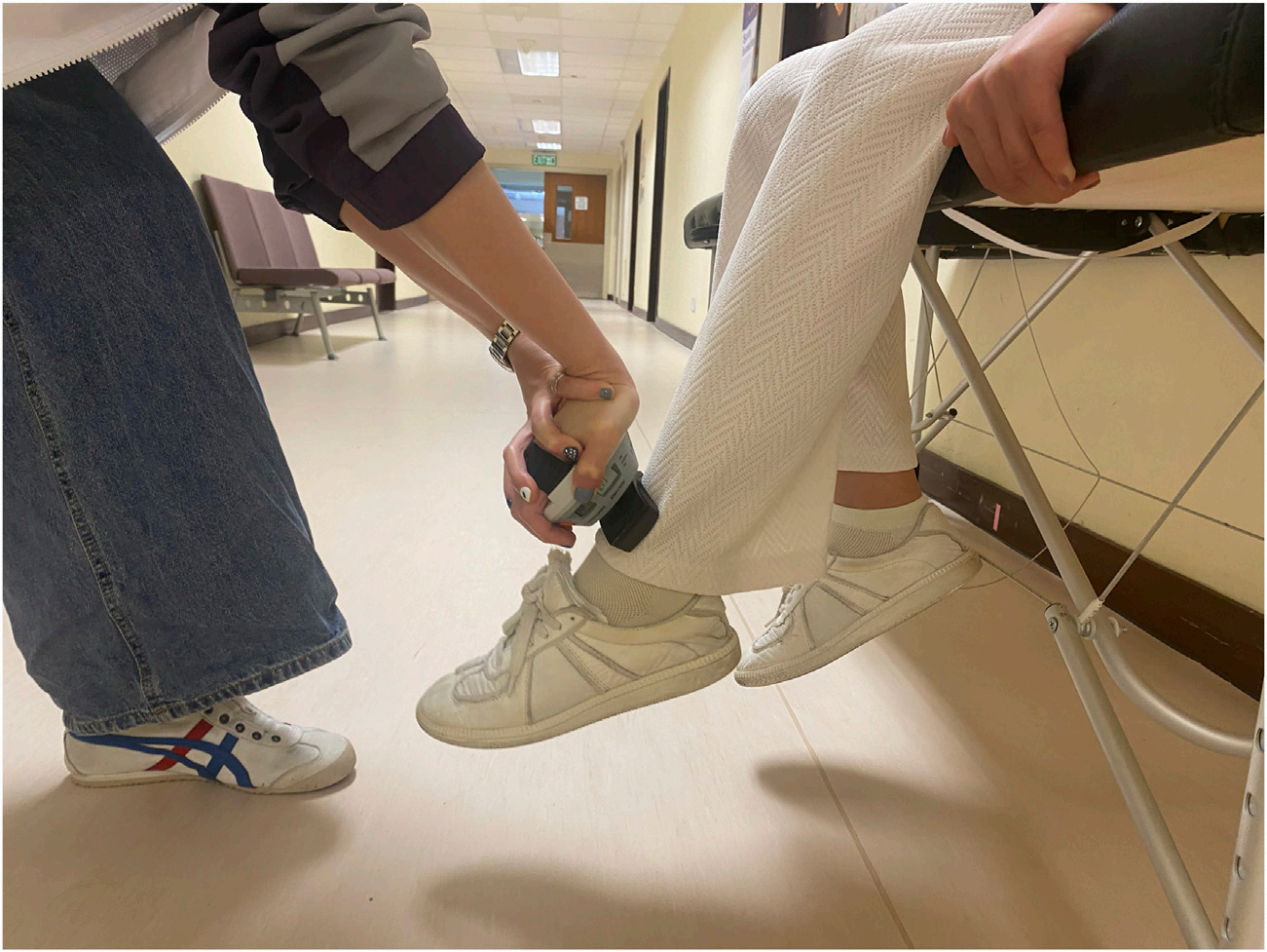
Isometric knee extension muscle strength test.

**FIGURE 2:**
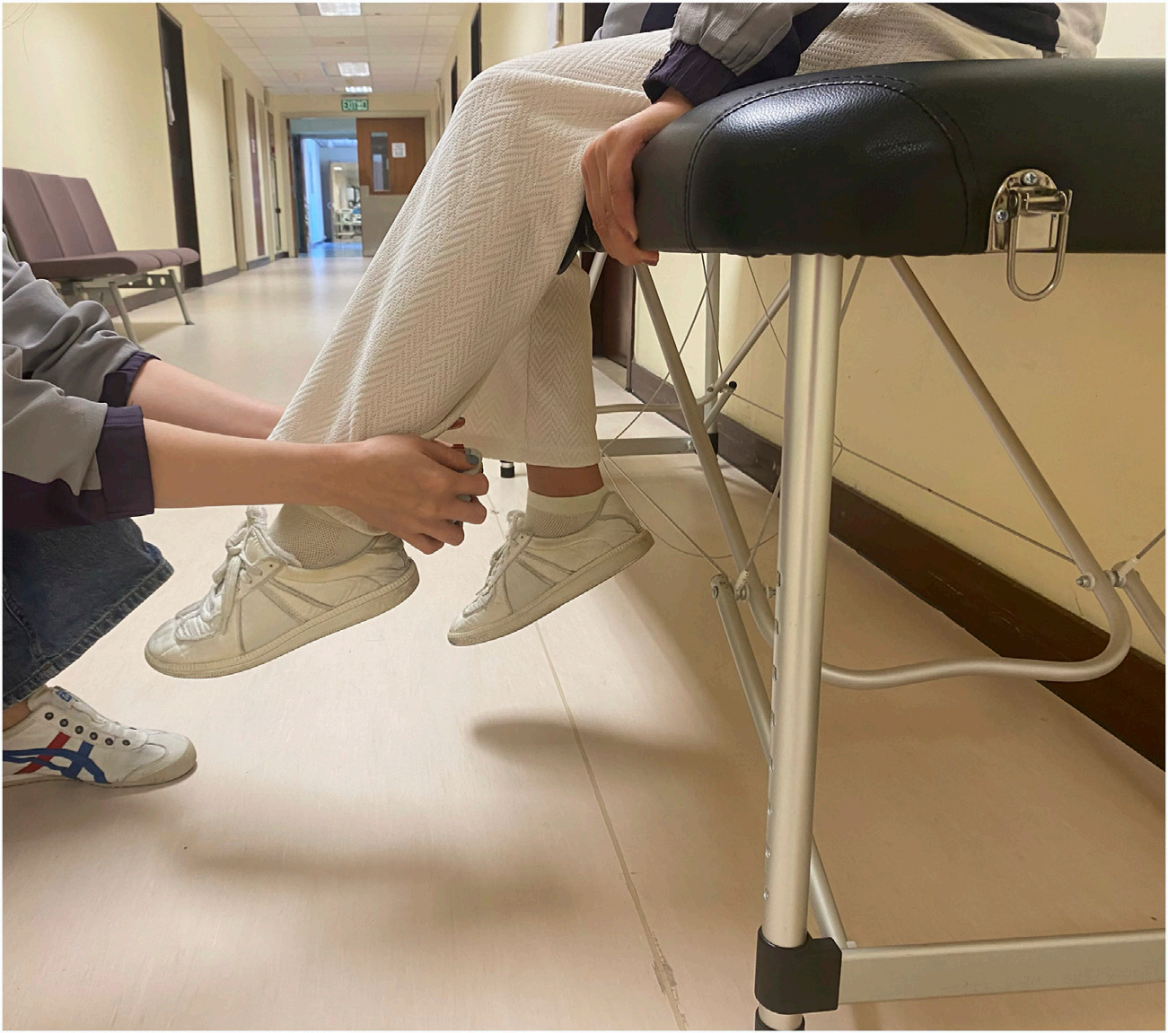
Isometric knee flexion muscle strength test.

Isokinetic dynamometry is the gold standard evaluation method for determining muscle strength. However, because of reduced proprioception and dysfunction of the quadriceps, the application of isokinetic measures in individuals with knee OA is dubious (Hortobágyi et al., 2004). Furthermore, isokinetic knee extension tests in an open kinetic chain may be harmful to the damaged cartilage and place significant pressure on the related ligaments and joints (Nisell et al., 1989; Kaufman et al., 1991). The hand-held dynamometer is a valid alternative tool to Biodex to measure isometric knee muscle strength (Grootswagers et al., 2022). It also has good reliability that same-day intrarater intraclass correlation coefficients (ICCs) ranged from 0.97 to 0.98. Interrater reliability ICCs ranged from 0.83 to 0.95 (Grootswagers et al., 2022). Maximal voluntary isometric knee muscle strength of the severe K/L grade side was assessed using a hand-held dynamometer (microFET 2; Hoggan Scientific, Salt Lake City, UT, United States). Participants were seated on an examination bed with their knees flexed to 60° and their feet off the ground and were asked to maintain the position. The hand-held dynamometer was positioned on the anterior aspect of the distal tibia, just superior to the malleoli. Participants were asked to grasp the examination bed with their hands for stabilization, and participants were instructed to extend or flex their knee “as hard as possible” into the hand-held dynamometer for 5 s, and the maximum force across the trial was recorded (See Figures 1, 2). Three testing trials were completed. The maximum knee extensor and flexor strength were calculated to obtain an overall muscle strength value (kgf) divided by the patient’s body mass.

#### Disease severity- radiological evaluation

Kellgren–Lawrence (KL) radiographic grading scale: Standing anteroposterior images were graded by two experienced physicians using the KL radiographic grading scale (Kellgren and Lawrence, 1957). The grades for this scale are as follows: 1 = questionable osteophytes, 2 = definite osteophytes without joint-space narrowing, 3 = definite osteophytes with moderate joint-space narrowing, and 4 = definite osteophytes with severe joint-space narrowing. In addition, grades 1 and 2 were classified as early-stage, and Grades 3 and 4 were classified as end-stage.

### Sample size

There is no golden standard approach to estimating the sample size requirements for risk prediction models currently. Tabachnick and Fidell (Tabachnick and Fidell, 2007) have provided a formula for calculating sample size requirements for multiple regression, considering the number of independent variables: n > 50 + 8m (where m = number of independent variables). We have chosen seven potential independent variables age, BMI, KL grade [early-stage (KL grade 2) and end-stage (KL grade 3–4)], durations of symptom, knee extension muscle strength, knee flexion muscle strength, and pain-KOOS score in this study. So, at least 106 knee OA patients were needed.

### Statistical analysis

Data were analyzed using SPSS version 29.0 (IBM Corp., Armonk, NY, United States). Descriptive statistics were used to describe demographic characteristics and key parameters of the cohort (i.e., mean values with SD). Spearman correlation coefficients were calculated between the physical function measures and other variables. Stepwise multiple linear regression analyses were performed using self-reported and performance-based physical function separately as dependent variables. Knee muscle strength, pain, age, BMI, durations of symptoms, and radiographic stage that were found significant correlations were entered into the stepwise multiple linear regression as independent variables to detect which factors determine physical function. A *p*-value less than 0.05 was considered statistically significant.

## Results

135 knee OA patients were assessed with 50 early-stage knee OA and 85 end-stage knee OA males. The demographic characteristics of the patients are summarized in Table 1. Self-reported physical function and performance-based physical function are presented in Table 2.

**TABLE 1:** Demographic, pathological, and muscle strength characteristics.

Parameters	
Age (years)	68.8 ± 6.2
n	135
Gender no. (%)	
Male	39 (28.9)
Female	96 (71.1)
Height (cm)	157 ± 8.3
Weight (kg)	65.4 ± 11.9
BMI (kg/m^2^)	26.4 ± 3.8
Durations of symptoms (years)	8.4 ± 5.9
K/L grade, no. (%)	
Grade 2	50 (37.0)
Grade 3–4	85 (63.0)
Knee extensor muscle strength (kgf/kg)	0.24 ± 0.08
Knee flexor muscle strength (kgf/kg)	0.18 ± 0.06
Pain-KOOS subscale	59.6 ± 15.6

**TABLE 2:** Results of self-reported and performance-based physical function.

Parameters	
PF-KOOS	69.8 ± 15.1
6MGS (m/s)	0.9 ± 0.2
5STS (s)	14.0 ± 5.5

Table 3 summarizes the correlation between the self-reported and performance-based physical function and other predictors. BMI and durations of symptoms did not indicate any significant correlation with either self-reported or performance-based physical function. For self-reported physical functioning, pain-KOOS (*r* ^2^ = 0.728, *p* < 0.01), knee flexor (*r* ^2^ = 0.313, *p* < 0.01), and extensor muscle strength (*r* ^2^ = 0.244, *p* < 0.01) all indicate a significant correlation. Age is significantly negatively associated with 6MGS (*r* ^2^ = −0.383, *p* < 0.01), while knee flexor (*r* ^2^ = 0.414, *p* < 0.01) and extensor (*r* ^2^ = 0.38, *p* < 0.01) muscle strength and pain-KOOS (*r* ^2^ = 0.303, *p* < 0.01) have a positive association with 6MGS. For 6MGS, knee extensor muscle strength (*r* ^2^ = −0.528, *p* < 0.01), knee flexor muscle strength (*r* ^2^ = −0.403, *p* < 0.01), pain-KOOS (*r* ^2^ = −0.234, *p* < 0.01) negatively correlated with 5STS, while age (*r* ^2^ = 0.197, *p* < 0.05) positively correlated with 5STS.

**TABLE 3:** Correlation coefficients.

Predictors	PF-KOOS	6MGS	5STS
Age (years)	−0.160	−0.383**	0.197*
BMI (kg/m^2^)	−0.090	−0.120	0.020
Durations of symptoms (years)	−0.099	−0.122	0.019
K/L grade	−0.105	−0.200*	0.121
Knee extensor muscle strength (kgf/kg)	0.244**	0.381**	−0.528**
Knee flexor muscle strength (kgf/kg)	0.313**	0.414**	−0.403**
Pain-KOOS subscale	0.728**	0.303**	−0.234**

**Correlation is significant at the 0.01 level (*p* < 0.01).

*Correlation is significant at the 0.05 level (*p* < 0.05).

The stepwise multiple linear regression models for all physical function measurements are summarized in Table 4. Pain intensity (β = 0.712, *p* < 0.001) and knee flexor muscle strength (β = 0.112, *p* = 0.420) were significantly associated with self-reported physical function in daily activities and contributed to 55.0% of the variance in KOOS-PF score, mostly explained by pain. BMI, durations of symptoms, and KL grade did not show a significant relation in stepwise regression models. Knee muscle strength, including knee extensor (5STS: β = 0.428, *p* < 0.001) and flexor muscle strength (6MGS: β = 0.367, *p* < 0.001), presented a significant association with performance-based physical function. In addition, low knee muscle strength and older age were related to limited functional performance.

**TABLE 4:** Stepwise multiple linear regression model.

Dependent variable	Predictors	*R* ^2^	β	P
PF-KOOS	Pain-KOOS subscale	0.539	0.712	<0.001
	Knee flexor muscle strength (kgf/kg)	0.550	0.112	0.042
6MGS (m/s)	Knee flexor muscle strength (kgf/kg)	0.173	0.367	<0.001
	Age	0.336	−0.389	<0.001
	Pain-KOOS subscale	0.378	0.209	0.004
5STS (s)	Knee extensor muscle strength (kgf/kg)	0.191	−0.428	<0.001
	Age	0.221	0.175	0.028

## Discussion

This study aimed to observe the predictors of self-reported and performance-based physical function in patients with knee OA by analyzing the impacts of demographic, pathological, and muscle impairment factors. Self-reported questionnaires measure what patients think they can do, while performance-based functional tests assess what patients “can do” (Abujaber et al., 2024). Self-reported questionnaires are used to gauge a patient’s opinion of their functional skills; nevertheless, the individual’s experience, emotions, recollections, and self-assurance all play a role in this process (Nielsen et al., 2016). Performance-based tests, on the other hand, assess a person’s true capacity to carry out a particular job; yet, they only represent a moment in time and are subject to the patient’s motivation (Fioravanti et al., 2012).

Our results show that pain intensity was the main predictor of self-reported physical function, and knee flexor muscle strength also contributed. However, knee extensor and flexor muscle strength were the primary determinants of performance-based measurements. Pain were also associated with performance-based measurements. Age significantly correlated with performance-based physical function, and it is the secondary but not main predictor for 6MGS and 5STS in this study. The severity of knee OA and BMI did not contribute to these two types of assessments.

Although knee OA is diagnosed and defined as loss of cartilage within the joint, knee muscle deficit may be the primary underlying cause of functional impairments (O Reilly et al., 1998) and the risk factor for disease onset (Øiestad et al., 2015) and other longitudinal evidence has shown that greater quadriceps strength (normalize to body mass) could protect against cartilage degradation (Amin et al., 2009). Compared to healthy age-matched older adults, knee OA patients were inclined to have a 25%–45% loss of knee extensor muscle strength and a 19%–25% loss of knee flexor muscle strength (Al-Johani et al., 2014). In our study, pain severity and knee flexion muscle strength were the most important determinants of self-reported physical function: a model including these variables accounted for a 55.0% variance in PF-KOOS scores. Knee flexion muscle strength also explained a 17.3% variance in the 6MGS, and knee extension muscle strength explained a 19.1% variance in the 5STS. The coefficient of the interaction variable shows that the knee flexion muscle strength that increases by 1 kgf/kg will raise the 6-meter gait speed by 0.367 m/s (β = 0.367) and increase self-reported physical function scores by 0.112 (β = 0.112). When knee extension muscle strength improves, 1 unit will improve performance 5STS by 0.428s for knee OA patients. Therefore, improving knee muscle strength is important to improve physical function in knee OA patients. We also found that knee muscle strength was negatively correlated with physical performance measurements and self-reported physical function and explained a significant proportion of performance-based functional limitations in knee OA patients. Alnahdi et al. (2014) concluded that in patients after total knee replacements, hip abductors and knee extensors strength were related to performed-based physical function but not to the self-reported measures. In another cross-sectional study, knee extension muscle strength was significantly associated with performance-based physical function (Tevald et al., 2016). Despite the fact that correlation analysis and measurement tools were used to find the relationships, the current study’s findings are consistent with the previous study. Pre-operative quadriceps strength can predict postoperative physical function up to a year following surgery (Mizner et al., 2005). Our results highlight the importance of knee muscle strength, especially in performance-based physical function. Rehabilitation should consider improving both quadriceps and hamstring muscle strength sufficiently to increase function and protect against the progression of knee OA.

Self-reported pain was the primary predictor of self-reported physical functioning and contributed to gait speed performance. Previous work in patient with knee or hip OA revealed that pain is a predictor for self-reported function (Stratford and Kennedy, 2006; Zeni et al., 2014). This result is also consistent with a former study that self-reported physical functioning is more affected by pain than performance-based functioning in female knee OA patients (Nur et al., 2018) although different pain measuring tools were used. In Nur’s study (Nur et al., 2018), VAS, the single-item index, was used, and we used multiple-item pain questionnaires (KOOS), which include various aspects of pain to calculate a total overall pain score. The experience of pain is multi-factorial, and factors such as patient depression also contribute an essential part in its manifestation in knee OA patients (McAlindon, 1999). One of our limitations is that we did not apply other psychological questionnaires to describe depression and anxiety conditions in knee OA patients. In addition to pharmacological, psychological, and surgical management for knee OA patients, a recent systematic review suggested that a 30%–40% increase in knee-extensor strength has been suggested to improve knee pain and disability (Bartholdy et al., 2017).

There is a widespread belief that a high discordance exists between clinical and radiographic knee OA (Hart et al., 1991). Although the more severe the radiographic osteoarthritis, the more likely there are accompanying symptoms, radiographic knee osteoarthritis is not a precise guide to the likelihood that knee pain or disability will be present (Bedson and Croft, 2008). We applied a secondary analysis by dividing our radiographic knee OA patients (KL grade 2 and above) into two groups early-stage (grade 2) and end-stage (grade 3–4) knee OA. In our study, although K/L grade has a weak negative association with 6WGS (*r* ^2^ = −0.200, *p* < 0.05). We did not find radiographic severity as a significant determinant of physical functioning, which is consistent with another study (Nur et al., 2018). The possible reason is that joint damage predisposes patients to limited walking ability, but it is not the root cause. In another cross-sectional study, Szebenyi et al. (2006) found that patients with structural changes in both the patellofemoral joint and tibiofemoral compartment rather than just one compartment have reduced function. We have based on an assessment of the tibiofemoral compartment only in our study, which would be the possible cause.

BMI did not correlate with physical function, which is inconsistent with other findings that obesity to be an important factor in self-reported physical function in knee OA patients. The possible reason is that the mean BMI (26.4) of our study population was noticeably low compared with similar literature. Creamer et al. (2000) suggested that BMI was one of the most important determinants of self-reported physical function; the mean BMI of the 69 knee OA patients was 31.4. In a sample of 1,272 African American and Caucasian individuals with a mean BMI of 28.3, obesity had independent effects on self-reported physical function. Another study concluded that BMI was one of the major attributes of self-reported physical function (Kauppila et al., 2009), the mean BMI of included 88 end-stage knee OA patients was 32.5. In one cross-sectional study, 110 female knee OA patients with a mean BMI of 31.0 were assessed for both self-reported and performance-based physical function. BMI only explained 0.3% of the variance of the self-reported physical function but did not contribute to performance-based physical function (Nur et al., 2018). More studies need to investigate whether BMI could predict worse self-reported and performance-based physical function in the Asian population.

In this study, we used performance-based and self-reported measures to assess physical functioning in patients with knee OA. To sum up, low knee muscle strength, age, and pain intensity significantly impacted performance-based limitations. In addition, pain intensity had more influence on self-reported limitations, and low muscle strength was also related to self-reported limitations. The severity of radiography knee OA and BMI are not the contributing factors to physical function. Therefore, added exercise-based intervention targeted at knee muscle strength would be beneficial for all stages of knee OA. For patients in the early stage of knee OA patients, improving knee muscle strength may prevent the disease from progressing. One study used the sensitive technique of magnetic resonance imaging (MRI) and a large sample size (*n* = 265 patients) to assess cartilage loss at a mean of 3.3 years. It also found that quadriceps weakness predicted whole knee and medial cartilage loss only in women (Lingard et al., 2000). For patients who are in the end stage, stronger knee muscle strength was also the modifiable predictor of physical function.

There are several limitations in this cross-sectional study. Firstly, we did not collect long-term follow-up data. Future studies could consider applying the longitudinal study design. Secondly, our study did not assess the knee range of motion, painkillers, and other psychological factors such as anxiety and depression.

### Clinical implications

Orthopedic surgeons should recommend knee OA patients pay attention to improving and maintaining their knee muscle strength, and it would be better to hand out a booklet with useful exercise guidelines. Physical therapists should design beneficial rehabilitation programs targeted at knee muscle strength. For primary care physicians, health lectures on the important role of knee muscle strength in the management of knee OA should be promoted.

### Future recommendations

Multiple interventions, such as exercise-based rehabilitation that focuses on strengthening knee muscles in both quadriceps and hamstring muscle strength could have beneficial effects in improving self-reported and performance-based function in knee OA patients. The different settings of duration, intensity, type, and delivery mode, such as supervised or home-based intervention of the high-quality randomized controlled trials, should be well designed to evaluate the short-term, sustained, and prolonged effects.

## Conclusion

In conclusion, pain intensity was the main risk factor of self-reported physical function, and knee flexor muscle strength contributed as well. The severity of knee OA, durations of symptoms and BMI did not contribute to physical function. However, knee extensor and flexor muscle strength were the main predictors of performance-based performance. Our results show that strengthening of weak knee muscles in both quadriceps and hamstring muscle strength and pain management should be considered a priority consideration in managing knee OA no matter whether people are in the early or end-stage knee OA.

## Scope statement

This cross-sectional study extended the knowledge of therapeutic targets in knee OA osteoarthritis, which is relevant to your special issue titled “New Insights on Bone, Cartilage and Degenerative Skeletal Diseases: From Molecular Mechanism to Clinical Therapy” under the Journal of Frontiers in Cell and Developmental Biology, Molecular and Cellular Pathology. We recommended paying attention to strengthening both knee extension and flexion muscle strength in patients with knee Osteoarthritis whatever stage they are in. It could inspire future studies on the different treatments according to different severity of knee Osteoarthritis patients.

## Data Availability

The original contributions presented in the study are included in the article, further inquiries can be directed to the corresponding author.

## Abbreviations

OA, Osteoarthritis; KOOS, Knee Injury and Osteoarthritis Outcome Score; 6MGS, 6-meter gait speed; 5STS, 5-time Sit-to-Stand test; BMI, body mass index; KL, Kellgren–Lawrence.
